# Expression Levels of MUC5AC and MUC5B in Airway Goblet Cells Are Associated with Traits of COPD and Progression of Chronic Airflow Limitation

**DOI:** 10.3390/ijms252413653

**Published:** 2024-12-20

**Authors:** Terezia Pincikova, Heta Merikallio, Ioanna Kotortsi, Reza Karimi, Chuan-Xing Li, Elisa Lappi-Blanco, Sara K. Lindén, Médea Padra, Åsa M. Wheelock, Sven Nyrén, Carl Magnus Sköld, Riitta L. Kaarteenaho

**Affiliations:** 1 Respiratory Medicine Unit, Department of Medicine Solna and Center for Molecular Medicine, Karolinska Institute, 171 76 Stockholm, Swedenasa.wheelock@ki.se (Å.M.W.);; 2 Stockholm CF-Center, Albatross, K56, Karolinska University Hospital Huddinge, 141 86 Stockholm, Sweden; 3 Research Unit of Biomedicine and Internal Medicine, University of Oulu, 902 20 Oulu, Finland; 4 Center of Internal Medicine and Respiratory Medicine, Medical Research Center Oulu, Oulu University Hospital, 902 20 Oulu, Finland; 5 Department of Respiratory Medicine and Allergy, Karolinska University Hospital, 171 76 Stockholm, Sweden; 6 Cancer and Translational Medicine Research Unit, Department of Pathology, Oulu University Hospital and University of Oulu, 902 20 Oulu, Finland; 7 Department of Medical Biochemistry and Cell Biology, Institute of Biomedicine, Sahlgrenska Academy, University of Gothenburg, 405 30 Gothenburg, Sweden; sara.linden@biomedicine.gu.se (S.K.L.);; 8 Department of Molecular Medicine and Surgery, Division of Radiology, Karolinska Institute, Karolinska University Hospital Solna, 171 76 Stockholm, Sweden

**Keywords:** COPD, immunohistochemistry, mucin, smoking, epithelium

## Abstract

Mucins 5AC (MUC5AC) and 5B (MUC5B) are the major mucins providing the organizing framework for the airway’s mucus gel. We retrieved bronchial mucosal biopsies and bronchial wash (BW) samples through bronchoscopy from patients with chronic obstructive pulmonary disease (*n* = 38), healthy never-smokers (*n* = 40), and smokers with normal lung function (*n* = 40). The expression of MUC5AC and MUC5B was assessed immunohistochemically. The mucin concentrations in BW were determined using the slot-blot technique. The immunohistochemical expression of MUC5AC and MUC5B was localized to goblet cells and submucosal glands. Smokers had higher MUC5AC and lower MUC5B goblet cell expression and higher concentrations of soluble MUC5AC in BW than never-smokers. The MUC5B expression in goblet cells correlated positively with expiratory air flows, diffusing capacity, and the dyspnoea score. Chronic bronchitis, emphysema, and the progression of chronic airflow limitation during a median follow-up time of 8.4 years were associated with higher MUC5AC and lower MUC5B expression in goblet cells. Sustainers, slow progressors, and rapid progressors of airflow obstruction differed in their MUC5B expression at baseline. Emphysema and bronchial wall thickening on CT at a follow-up visit were associated with lower MUC5B expression at baseline. Our findings strengthen the hypothesis that MUC5AC and MUC5B are yet another contributing factor to smoking-associated lung disease progression.

## 1. Introduction

Chronic obstructive pulmonary disease (COPD) is a disease characterized by chronic respiratory symptoms due to abnormalities in the airways and alveoli that cause persistent, often progressive, airway obstruction. In the appropriate clinical context, the presence of non-fully reversible chronic airflow limitation after bronchodilation confirms the COPD diagnosis [1]. Chronic mucus hypersecretion is a common feature of COPD, and mucus plugging of the airways is related to airflow obstruction and is also a prognostic marker of mortality [2,3]. The major structural components of respiratory mucus are the gel-forming mucins (MUCs) MUC5AC and MUC5B [4]. In smokers, goblet cell hyperplasia and hypertrophy, as well as abnormal patterns of mucin gene expression, have been described [5]. It is believed that a general overproduction of mucins contributes to morbidity and mortality in COPD [6]. Recently, it has become increasingly recognized that a large number of different mucins add to the complexity of the expression and function of these macromolecules [7]. Long-term smoking induces enhanced epidermal growth factor receptor and MUC5AC expression [8]. Accordingly, there is an up-regulation of MUC5AC in the small airway epithelium of smokers associated with a coordinated up-regulation of MUC5AC-associated core gene expression pattern [9]. Moreover, cytokines, oxidative stress, and endoplasmic reticulum stress stimulate MUC5AC production in airway epithelium [10,11,12]. In contrast, MUC5B deficiency in mice caused chronic infection by multiple bacterial species and inflammation that failed to resolve normally [13], which suggests a specific role for MUC5B. In line with that, airway wall thickness increased with MUC5AC mRNA overexpression in severe neutrophilic asthma. However, the expression of MUC5B was decreased, probably resulting in poor mucociliary clearance in the airways [14]. Accordingly, the congenital absence of MUC5B in humans recently defined a new category of genetic respiratory disease associated with sinus disease, impaired mucociliary clearance, and lung function impairment [15]. To our knowledge, the relationship between the MUC5AC and MUC5B immunohistochemical expression in airway mucosa and lung function in humans has not yet been studied. In addition, current knowledge of the immunohistochemical protein expression of MUC5AC and MUC5B in human airways on a cellular level is limited. Caramori et al. examined MUC5AC and MUC5B expression in bronchial rings retrieved from subjects undergoing bronchial ring resection for a solitary peripheral neoplasm [16]. Ma et al. examined MUC5AC expression in eighteen patients undergoing lung resections for a solitary peripheral carcinoma [17]. Data on MUC5AC and MUC5B expression in goblet cells of the large airway mucosa in healthy never-smokers, smokers with normal lung function, and smokers with COPD are missing.

We hypothesized that the immunohistochemical expression of MUC5AC and MUC5B in human large airways would differ among never-smokers, smokers, and COPD patients and that this may also be reflected by alterations in secreted MUC5AC and MUC5B concentrations. We also hypothesized that these mucins are related to lung function impairment and the progression of chronic airflow limitation over time. To test this hypothesis, we investigated bronchial mucosal biopsies and bronchial wash (BW) samples from never-smokers, smokers with normal lung function, and COPD patients. We also performed spirometry and computed tomography (CT) scans of the chest after a median follow-up period of 8.4 years.

## 2. Results

### 2.1. Expression of MUC5AC and MUC5B in the Large Airway Epithelium at Baseline

**Figure 1:**
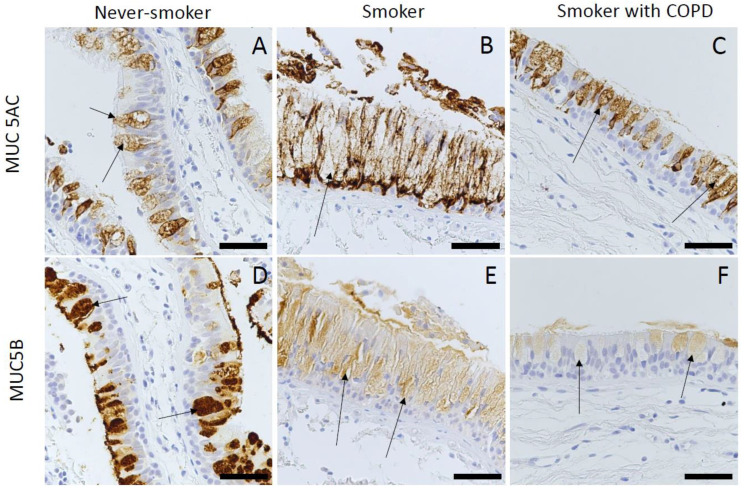
Representative images of the immunohistochemical staining for MUC5AC and MUC5B in bronchial biopsy samples retrieved from large airways. Scores of the expression were assigned as negative (0), faint (1), moderate (2), strong (3), or very strong (4). MUC5AC expression in goblet cells in a never-smoker with normal lung function (**A**), a smoker with normal lung function (**B**), and a smoker with COPD (**C**). MUC5B expression in goblet cells in a never-smoker (**D**), a smoker with normal lung function (**E**), and a smoker with COPD (**F**). The length of the scale bar in the immunohistochemical images is 50 µm. Black arrows (→) show goblet cells.

The immunohistochemical MUC5AC expression was localized to goblet cells, and all goblet cells stained positive. The intensity of the expression varied from weak to strong. The MUC5B expression was localized to both goblet cells and submucosal glands, but since many of the biopsies lacked the submucosal gland area, the MUC5B expression in submucosal glands was not included in the analyses (Figure 1A–F).

### 2.2. Effects of Smoking on the Expression of MUC5AC and MUC5B at Baseline

**Figure 2:**
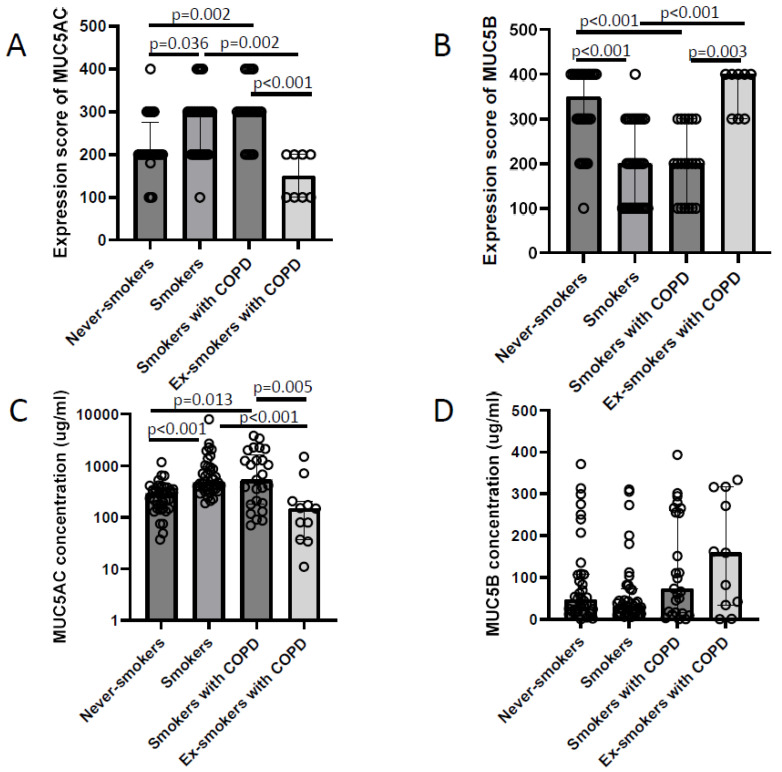
MUC5AC expression (**A**) and MUC5B expression (**B**) in goblet cells in never-smokers, smokers with normal lung function, smokers with COPD, and ex-smokers with COPD. MUC5AC concentration (**C**) and MUC5B concentration (**D**) in BW in never-smokers, smokers with normal lung function, smokers with COPD, and ex-smokers with COPD. Kruskal–Wallis Test was used in all. Bars represent the median with interquartile range.

The immunohistochemical MUC5AC expression was higher in smokers compared to never-smokers. In addition, ex-smokers with COPD expressed less MUC5AC than smokers with COPD and smokers with normal lung function (Figure 2A). The intensity of MUC5B expression in goblet cells varied between the subjects and was higher in never-smokers compared to smokers. Moreover, MUC5B expression was higher in ex-smokers with COPD than in smokers (Figure 2B).

In agreement with the immunohistochemical results, the concentration of MUC5AC in BW samples was higher in smokers compared to never-smokers. Smokers with COPD had a higher concentration of MUC5AC in BW than ex-smokers with COPD (Figure 2C). However, the concentration of soluble MUC5B in BW did not differ among the study groups (Figure 2D).

**Figure 3:**
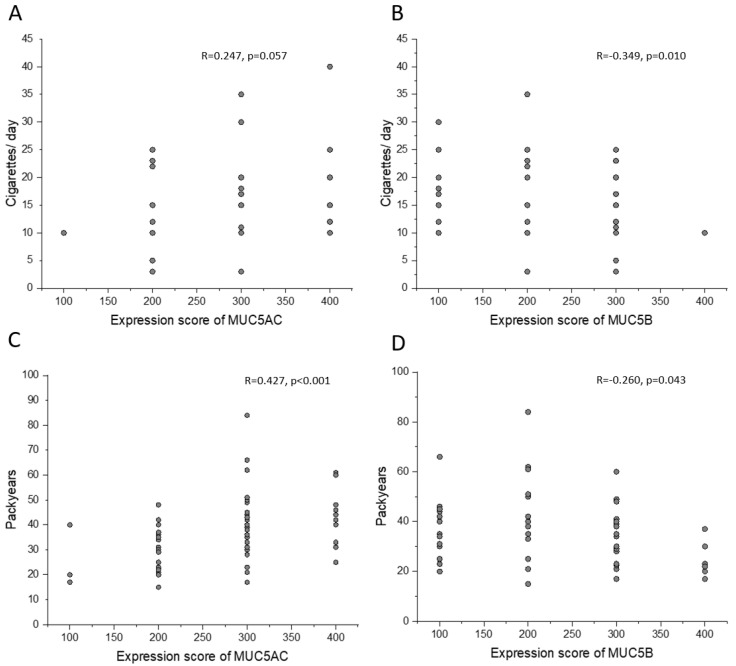
Associations between smoking exposure and the immunohistochemical expression for MUC5AC and MUC5B. Association between current cigarette consumption and MUC5AC and MUC5B immunohistochemical expression in goblet cells in smokers with normal lung function and smokers with COPD (**A**,**B**). Association between smoking history (pack-years) and MUC5AC expression (**C**) and MUC5B expression (**D**) in goblet cells in smokers with normal lung function, smokers with COPD, and ex-smokers with COPD. Spearman correlation analyses in all.

In smokers, both with COPD and normal lung function, current cigarette consumption tended to correlate with MUC5AC expression and correlated modestly negatively with MUC5B expression in goblet cells (Figure 3A,B). Also, smoking history (pack-years) in smokers with normal lung function and smokers with COPD, as well as in ex-smokers with COPD, correlated positively with MUC5AC expression in goblet cells (Figure 3C) and negatively with the immunohistochemical MUC5B expression in goblet cells (Figure 3D). The results were confirmed by exhaled carbon monoxide measured before bronchoscopy (Supplementary Figure S1A,B).

### 2.3. Association of MUC5AC and MUC5B Expression with Lung Function, Emphysema, and Chronic Bronchitis at Baseline

The immunohistochemical MUC5B expression in goblet cells correlated positively with postbronchodilator forced expiratory volume in one second (FEV_1_), forced vital capacity (FVC), slow vital capacity (VC), Hb-corrected diffusing capacity, and dyspnoea score, assessed by Chronic Respiratory Disease Questionnaire at baseline (Table 1 and Supplementary Figure S2A).

**Figure 4:**
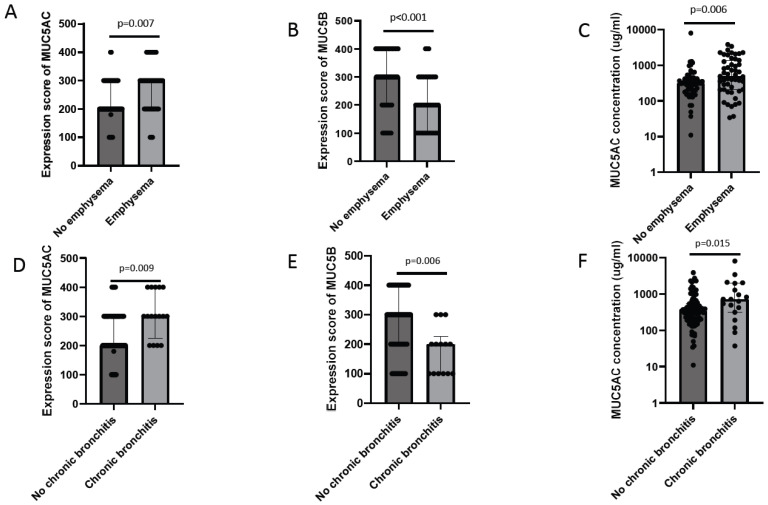
Differences in MUC5AC expression, MUC5B expression, and MUC5AC concentration in BW in subjects with or without emphysema (**A**–**C**) and in subjects with or without chronic bronchitis (**D**–**F**) at baseline. The immunohistochemical MUC5AC expression (**A**,**D**) and MUC5B expression (**B**,**E**) in goblet cells, and MUC5AC concentration in BW samples (**C**,**F**). Mann–Whitney U Test in all. Bars represent the median with interquartile range.

In addition, the MUC5AC concentration in BW correlated negatively, although weak, with diffusing capacity (Supplementary Figure S2B). In line with this, patients with emphysema had higher MUC5AC expression in goblet cells and higher MUC5AC concentration in BW fluid, as compared with patients without emphysema. On the contrary, MUC5B expression in goblet cells was lower in individuals with emphysema at baseline (Figure 4A–C). Patients with chronic bronchitis had higher immunohistochemical MUC5AC expression, higher MUC5AC concentration in BW, and lower immunohistochemical MUC5B expression in goblet cells, as compared with patients without chronic bronchitis at baseline (Figure 4D–F). There were no differences in the immunohistochemical expression of MUC5AC or MUC5B in goblet cells or their concentration in BW between men and women(p = 0.36, p = 0.31, p = 0.90 and p = 0.74, respectively).

### 2.4. Association of MUC5AC and MUC5B Expression with Prospective Follow-Up Outcomes

**Figure 5:**
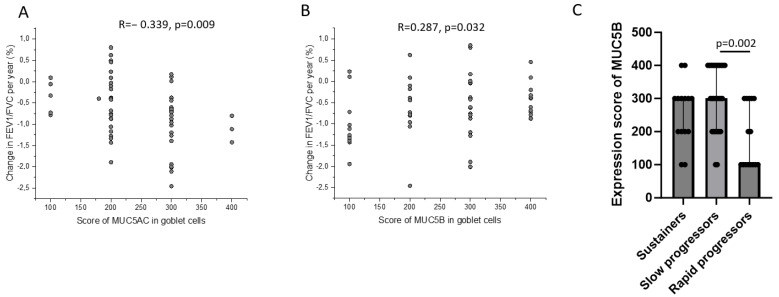
Association between change in FEV1/FVC ratio per year in the follow-up study cohort (*n* = 74) and the immunohistochemical expression of MUC5AC (**A**) and MUC5B (**B**) assessed in biopsies of large airway mucosa retrieved at baseline. The immunohistochemical MUC5B expression (**C**) in goblet cells at baseline, assessed in sustainers, slow progressors, and rapid progressors. Sustainers, slow progressors, and rapid progressors were defined as subjects with a change in the FEV1/FVC ratio during the follow-up time as above the upper quartile, between the lower and the upper quartile, and below the lower quartile, respectively. Spearman correlation analyses in (**A**,**B**). Kruskal–Wallis Test in (**C**). Bars represent the median with interquartile range.

A change in the FEV_1_/FVC ratio per year during a median follow-up time of 8.4 years correlated negatively with the immunohistochemical expression of MUC5AC and positively with the immunohistochemical expression of MUC5B in goblet cells at baseline (Figure 5A,B). Next, we divided the study subjects in the follow-up study into sustainers, slow progressors, and rapid progressors. Sustainers, slow progressors, and rapid progressors were defined as subjects with a change in the FEV_1_/FVC ratio during the follow-up time as above the upper quartile, between the lower and the upper quartile, and below the lower quartile, respectively. Sustainers, slow progressors, and rapid progressors differed in MUC5B expression in goblet cells at baseline (Figure 5C).

**Figure 6:**
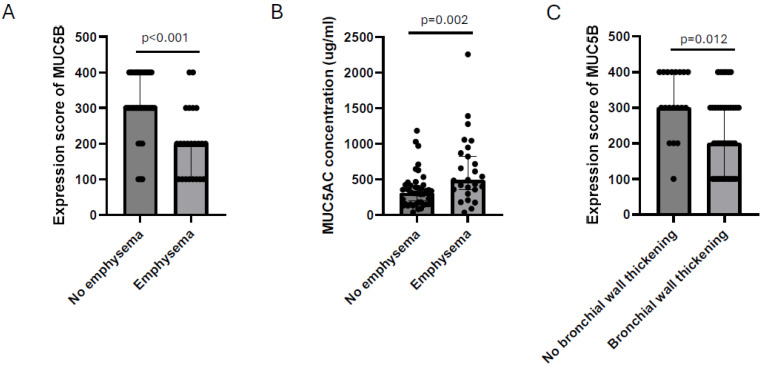
The immunohistochemical expression of MUC5B in goblet cells (**A**) and the concentration of soluble MUC5AC in BW (**B**) at baseline in subjects who had emphysema on CT scan at the end of follow-up compared with subjects who did not have emphysema on CT scan at the end of follow-up. The immunohistochemical expression of MUC5B in goblet cells (**C**) at baseline in subjects who had signs of bronchial wall thickening on CT scan at the end of follow-up compared with subjects who did not have signs of bronchial wall thickening on CT scan at the end of follow-up. Mann–Whitney U Test in all. Bars represent the median with interquartile range.

Consistent with lung function, subjects who had emphysema on CT scan at follow-up visit had lower MUC5B expression in goblet cells and higher concentration of soluble MUC5AC in the bronchial wash at baseline (Figure 6A,B). Moreover, subjects who had bronchial wall thickening on CT scan at follow-up visit had significantly lower MUC5B expression in goblet cells at baseline (Figure 6C).

Accordingly, smoking status at the follow-up visit (never-smoker, ex-smoker, or smoker) was associated with the immunohistochemical expression of MUC5AC and MUC5B in goblet cells at baseline (Supplementary Figure S3A,B). The number of years that have passed since smoking cessation correlated positively with the immunohistochemical expression of MUC5B in goblet cells at baseline (Supplementary Figure S3C).

## 3. Discussion

Here, we demonstrate that MUC5AC and MUC5B are both expressed in goblet cells in the large airway mucosa and that the immunohistochemical expressions are associated with smoking exposure, emphysema, chronic bronchitis, and with progression of chronic airflow limitation during a follow-up time of close to one decade. In addition, the goblet cell expression of MUC5B correlated positively with expiratory air flows. We speculate that these mucins might have two different underlying mechanisms regulating their expression in goblet cells or two different functions.

Recently, there has been a shift from the paradigm of repressing generally increased mucin expression to targeting the regulation of specific mucins [7]. Studies indicated that MUC5B is required for mucociliary clearance, whereas MUC5AC does not contribute to this process [13]. In murine models, MUC5B deficiency led to mucous obstruction of the airways and increased risk of inflammation and infection [13]. In contrast, up-regulation of MUC5AC was implicated in the pathophysiology of cystic fibrosis, asthma, and COPD, as reviewed in [18]. In line with this, in our cohort, high MUC5AC and low MUC5B expression were associated with smoking exposure as well as with unfavorable traits of COPD, such as chronic bronchitis and emphysema. A recent study demonstrated that there is a high fraction of secretory granules containing both MUC5AC and MUC5B and, therefore, the authors speculated that it may be unlikely that their secretion can be differentially controlled as a therapeutic strategy [19]. In our cohort, smoking exposure, chronic bronchitis, and emphysema were associated with high MUC5AC expression but with low MUC5B expression in goblet cells of the large airway mucosa. We speculate that differential control of these mucins at the level of goblet cells before they are secreted may be a potential therapeutic strategy instead of trying to control their secretion. Further studies are needed to elucidate this.

Current smoking, even in the absence of COPD, is associated with increased goblet cell density and mucin volume density [20]. Long-term smokers displayed a higher level of large MUC5AC complexes in the peripheral airway lumen than healthy non-smokers [21]. Acrolein, a component of cigarette smoke, induces MUC5AC expression via an initial ligand-dependent activation of the epidermal growth factor receptor, mediated by a disintegrin and metalloprotease 17 and matrix metallopeptidase 9 [22]. In airway epithelial cells, cigarette smoke extract increased toll-like receptor 3-stimulated MUC5AC production, mainly via extracellular signal-regulated kinase signaling [23]. The literature also suggests that there are differences in mucin amounts and properties between smokers with and without COPD [24]. Results of our study indicate that smoking might indeed affect mucin expression. In our cohort, the total smoking exposure (pack-years) correlated positively with the immunohistochemical expression of MUC5AC and negatively with the immunohistochemical expression of MUC5B. We confirmed this by investigating exhaled carbon monoxide as a surrogate marker of cigarette smoke exposure. In line with this, the immunohistochemical expression of MUC5AC and MUC5B in goblet cells differed between never-smokers and smokers and between ex-smokers and smokers. However, smokers with normal lung function had similar MUC5AC and MUC5B expression in goblet cells as smokers with COPD. This indicates that MUC5AC and MUC5B expression in goblet cells is associated with smoking exposure independently of the presence of COPD. These findings are in line with the results of our previous study, where we found that smoking history and the presence of chronic bronchitis were associated with both cellular and soluble MUC1 in human airways [25]. We also showed that smokers had higher messenger-RNA levels of MUC3A and MUC3B than non-smokers [26], despite the fact that these molecules undergo major post-translational regulation. Moreover, we have found an alteration of 50% of the proteome in alveolar macrophages in smokers compared to non-smokers, potentially associated with dysfunctional mucociliary escalator functions [27]

Interestingly, while the immunohistochemical MUC5AC expression and the concentration of MUC5AC in BW correlated positively with smoking exposure, MUC5B did not, suggesting there might be various underlying regulatory pathways or functions of these mucins. Several other previous studies point in the same direction. For example, in mice, MUC5B was required for controlling infections in the airways [13]. We speculate that smoking might lead to decreased MUC5B expression in goblet cells, thereby impairing infection control mechanisms and increasing susceptibility to acute and chronic infections, which is one of the characteristic clinical features of COPD. Consistent with this hypothesis, MUC5B expression in goblet cells correlated positively with expiratory air flows as well as with diffusing capacity. Moreover, a change in the FEV_1_/FVC ratio per year, as a measure of the progression of chronic airflow limitation, correlated negatively with the immunohistochemical expression of MUC5AC and positively with the immunohistochemical expression of MUC5B in goblet cells, assessed at the initial study visit. Collectively, these results suggest that MUC5AC and MUC5B might be involved in COPD pathogenesis or may be biomarkers of lung disease. In line with this, MUC5B was shown to be essential for maintaining normal lung function in mice [28]. In humans, the congenital absence of MUC5B recently defined a new category of genetic respiratory disease associated with sinus disease, impaired mucociliary clearance, and lung function impairment [15]. Moreover, the sputum MUC5AC and MUC5B concentrations were associated with common genetic variants, and the top locus for MUC5B might influence COPD phenotypes, in particular chronic bronchitis [29]. Accordingly, genes elevated in never-smokers with a more rapid decline in FEV_1_ were significantly enriched in mucin-related genes [30]

In our cohort, the immunohistochemical MUC5B expression in goblet cells was positively correlated with diffusing capacity, dyspnoea score, and with expiratory air flows at the baseline visit. Moreover, it was associated with a change in the FEV1/FVC ratio during the median follow-up time of 8.4 years. Also, the signs of emphysema at the follow-up visit were associated with lower MUC5B expression, higher MUC5AC expression, and higher MUC5AC concentration in BW at baseline. Similarly, chronic bronchitis, which is recognized as an unfavorable clinical trait of COPD, was associated with lower MUC5B expression, higher MUC5AC expression, and higher MUC5AC concentration in BW. Interestingly, the number of years that have passed since smoking cessation correlated positively with the immunohistochemical expression of MUC5B in goblet cells at baseline. This indicates that the mechanisms mediating the effect of smoking and regulating the levels of MUC5B in the large airways might be at least partially reversible. Future studies elucidating the underlying mechanisms regulating the MUC5B expression are warranted. We speculate that high MUC5AC expression and low MUC5B expression might be biomarkers primarily reflecting smoking exposure and thus associated with the risk of developing emphysema and bronchial wall thickening and with the progression of chronic airflow limitation in the long run. Eventually, this might lead to the development of COPD. The data also indicate that MUC5B expression may increase after smoking cessation, and we hypothesize that this might be part of the restorative physiological processes ongoing after the smoking individuals quit smoking. These novel findings need to be confirmed in a larger cohort, and further studies are needed to illuminate the role MUC5B plays in the physiology of the human lung.

Kesimer et al. demonstrated that the concentrations of both MUC5AC and MUC5B in induced sputum of current smokers or ex-smokers with severe COPD were higher than in never-smokers [31]. In contrast to the study by Kesimer et al. [31], we found that the immunohistochemical expression of MUC5B in goblet cells was lower in subjects with chronic bronchitis and that the MUC5B concentration in BW was not associated with chronic bronchitis. Our results cannot be directly compared with the findings of Kesimer et al. as we looked at the immunohistochemical expressions of mucins in goblet cells of the large airways and not in induced sputum. Radicioni et al. found that increased MUC5AC concentrations in induced sputum were more reliably associated with manifestations of COPD than MUC5B concentrations [32]. Tajiri et al. found that the ratio of induced sputum MUC5AC to MUC5B levels tended to be higher in patients with mild asthma than in controls and the sputum MUC5B levels were negatively correlated with airway reactivity [33]. All these previous studies did not examine airway mucosal biopsies and, therefore, did not examine mucin protein expression at the cellular level. However, many of our biopsies lacked the submucosal gland area. Therefore, we were unable to evaluate mucin expressions in the subepithelial area. Thus, the MUC5B expression in the submucosal glands was not included in our analysis. We speculate that the MUC5B expression in the submucosal glands might contribute to the MUC5B content in sputum and that this might partially explain the fact that the previously published papers on MUC5B in induced sputum contradict our findings. Collectively, studies designed to explore how MUC5B concentration in airway fluids relates to its immunohistochemical protein expression in airway mucosa appear to be warranted.

A limitation of our study is the lower number of participants in the follow-up study. On the other hand, the strengths of our study are the relatively long follow-up time of median 8.4 years, a robust baseline cohort, detailed radiological assessment by CT scan, and the fact that we had good-quality biopsies of the large airway mucosa combined with sensitive immunohistochemical analysis that enabled us to evaluate MUC5AC and MUC5B expression in goblet cells at the cellular protein level.

## 4. Materials and Methods

### 4.1. Study Subjects

The subjects included in this study were part of the Karolinska COSMIC (Clinical & Systems Medicine Investigations of Smoking-related Chronic Obstructive Pulmonary Disease) cohort (www.ClinicalTrials.gov/ct2/show/study/NCT02627872, accessed on 12 November 2024), as described previously [34]. Briefly, we investigated healthy never-smokers (“never-smokers”), current smokers with normal lung function (“smokers”), and COPD patients with postbronchodilator forced expiratory volume in 1 s/forced vital capacity (FEV_1_/FVC) < 0.7 and forced expiratory volume in 1 s (FEV_1_) 50–100% predicted (“COPD patients”). The study subjects were recruited through advertisements during the years 2007–2011 from the general Stockholm population aged 45–65 years. The groups were matched in terms of age, sex, smoking history (>10 pack-years), and current smoking habits (smoking > 10 cigarettes/day past 6 months). The latter was verified by exhaled carbon monoxide [35]. The exclusion criteria for all study groups included a diagnosis of asthma or allergy, a positive screening test for the common airborne allergens ”Phadiatop”, treatment with immunomodulatory medications or theophylline, current pregnancy or planning pregnancy, alcohol or drug abuse, other significant comorbidities as assessed by the admitting physician, and significant pathology on CT scan, X-ray, ECG, or in blood lab tests. Local steroids in the form of eye drops or nasal sprays were allowed. Bronchodilators, either regularly or as needed, were allowed. The subjects were free from airway infections for at least 4 weeks and free from airway infections treated with antibiotics for at least 3 months. Moreover, smokers and COPD patients were free from lung exacerbations for the past 3 months. COPD patients did not use steroids for inhalation for the past 3 months and did not have any other lung disease than COPD as assessed by the admitting physician. Ex-smokers have been completely free from smoking for a minimum of 2 years. The study was approved by the Stockholm Regional Ethics Board (No. 2006-959-31/1; 2006-10-27), and all participants gave informed written consent.

### 4.2. Baseline Visit

All subjects underwent clinical examination, chest CT scan, and spirometry, including diffusing capacity for carbon monoxide. The clinical characteristics of the baseline cohort are shown in Table 2. Emphysema was evaluated on inspiratory CT scans by two experienced reviewers (SN and ReKa) and considered to be present when more than 5% of the whole lung parenchyma was occupied by emphysematous changes. Bronchial wall thickening was considered to be present when both central bronchi and peripheral wall thickening were present simultaneously. The dyspnoea score was assessed by Chronic Respiratory Disease Questionnaire. Chronic bronchitis was considered to be present if the individuals reported productive cough for at least 3 months per year during the last 2 years. All study subjects (never-smokers, smokers with normal lung function, and COPD patients) were assessed for the presence of emphysema (yes/no) and chronic bronchitis (yes/no). Several subjects had both emphysema and chronic bronchitis.

Bronchoscopy was performed as previously described [36]. The bronchial mucosal biopsy specimens were taken using pulmonary biopsy forceps with smooth edge jaws (Radial Edge^®^ Biopsy Forceps, Boston Scientific, Boston, MA, USA). Four to six biopsies were taken from each subject, and they were collected from the lobar or segmental carinae of the upper lobes or the apical segment of the lower lobes. The BW samples were taken by instilling 10 mL of sterile phosphate-buffered saline at 37 °C into a segmental bronchus in the right upper lobe, after which the fluid was gently suctioned back. The samples were frozen without filtration, as described previously [25].

### 4.3. Follow-Up Visit

The COSMIC follow-up study was approved by the Stockholm Regional Ethics Board (No. 2016/327-31/4), and all participants gave informed written consent. Altogether, 74 individuals (63%) of the original cohort participated in a follow-up study. The clinical characteristics of the follow-up cohort are shown in Table 3.

After a median follow-up time of 8.4 years, the study subjects underwent a new spirometry and chest CT scan. The percent of predicted values was calculated using the ECCS reference equations. The progression of chronic airflow limitation was assessed as a change in the FEV1/FVC ratio (%) per year. The sustainers were defined as subjects with a change in the FEV_1_/FVC ratio (during the follow-up period) above the upper quartile. The slow progressors were defined as subjects with a change in the FEV1/FVC ratio between the lower and the upper quartile. The rapid progressors were defined as subjects with a change in the FEV1/FVC ratio below the lower quartile. The chest CT scan was assessed by the same two experienced reviewers (SN and ReKa) as at the baseline visit.

### 4.4. Immunohistochemical Staining

All biopsies were immediately formalin-fixed and embedded in paraffin. The tissue samples were stained with hematoxylin-eosin, and the representativeness of all biopsies was evaluated. Two representative tissue blocks from each subject were selected for immunohistochemical analyses. The immunohistochemical staining was performed in serial, consecutive sections. Alcian-Blue periodic acid-Schiff (AB-PAS) staining was performed for phenotyping goblet cells. Four μm thick sections were cut, deparaffinized with xylene, and rehydrated in a descending ethanol series. The primary antibodies used in the immunostaining were designed for formalin-fixed paraffin-embedded tissues. All antibodies were stained with a DAKO REAL EnVision-kit from Dako (Dako, Glostrup, Denmark). Before the application of the primary antibodies for MUC5AC (Clone CLH2, Novocastra, Leica biosystems, Newcastle, UK) and MUC5B (clone H-300, Santa Cruz, CA, USA), the sections were heated in a microwave oven in 10 mM citrate buffer, pH 6.0, for 10 min. After overnight incubation at +4 °C with the primary antibody, a biotinylated secondary HRP Rabbit/mouse -antibody (Dako, Envision) was used. In all the immunostainings, the color was developed with diaminobenzidine, after which the sections were lightly counterstained with hematoxylin. Antibodies used in the immunohistochemical staining are shown in Supplemental Table S1.

In the evaluation of the immunohistochemical samples, cytosolic expression was considered significant. The intensity of immunostaining was assessed as zero (negative) to four (strong positive), and the extent of the positive staining was estimated from 0% to 100% in each cell type. The score for each antibody correlated total intensity with extent, resulting in a total score with a range between 0 and 400, as published previously [25,26]. The evaluation was performed blinded to the clinical information of the study subjects by an experienced researcher (HM). Forty percent of the samples were also evaluated by a pulmonary pathologist (RiKa). According to Cohen’s kappa (Ƙ) coefficient, the intra-class correlation between the two assessments was 0.70 and categorized as substantial [37]. The representative stainings are shown in Figure 1A–F.

### 4.5. Quantification of Soluble MUC5AC and MUC5B

Secreted MUC5AC and MUC5B were measured from the bronchial wash samples using quantitative immunodetection on slot-blotted samples. A complete protease inhibitor cocktail (p8340, Sigma-Aldrich, Stockholm, Sweden) was added to the BW samples during thawing (10 µL to 1 ml of sample). The samples were diluted 1/100 in reduction buffer (6 M GuHCl, 5mMEDTA, 0.1 M Tris/HCl, pH 8.0). For the MUC5B detection, the samples were reduced using 2 mM 1,4-dithiothreitol in 0.1 M Tris–HCl buffer, pH 8.0, containing 6 M guanidinium chloride and 5 mM sodium EDTA, at 37 °C for 1h and then alkylated in 5 mM iodoacetamide at room temperature for 1 h to expose the epitope. 100 μL of each sample was loaded onto a PVDF-FL membrane (Millipore, Bedford, MA, USA) using a Minifold–II Slot Blot apparatus (Schleicher & Schuell Bioscience, Hahnestrasse 3, Dassel, Germany). In addition, nine serial dilutions of the mucin standards (purified human MUC5AC isolated from the stomach and MUC5B isolated from the lung) were also loaded. The vacuum was applied to attach the mucin to the membrane. The membranes were then dried for 1 h, pre-wetted briefly in 100% methanol, rinsed with ultrapure water, and incubated in phosphate-buffered saline (0.14 M NaCl, 0.0027 M KCl, 0.010 M PO_4_^3^) for 10 min. Unspecific binding was blocked by incubating in Odyssey blocking buffer (LI-COR Biosciences) for 1 h at 22 °C. The membranes were then incubated with rabbit serum against MUC5B (MUC5B-2) diluted 1:16,000 or with a mouse monoclonal antibody against MUC5AC (clone 45MI, Sigma-Aldrich St. Louise, MO, USA) diluted 1:1000 in Odyssey blocking buffer, containing 0.1% Tween 20, overnight at 4 °C with gentle shaking. The membranes were washed four times for 5 min each at 22 °C in phosphate-buffered saline containing 0.1% Tween 20. Thereafter, the membranes were incubated with goat anti-mouse IR dye 800 or goat anti-rabbit IR dye 680 secondary antibody (LI-COR, Biosciences, NE, USA) diluted 1:10,000 in blocking buffer containing 0.1% Tween-20 and 0.01% SDS for 30 min in the dark at 22 °C. The membranes were washed (4 × 5 min) in phosphate-buffered saline containing 0.1% Tween 20, and the blots were imaged using the Odyssey infrared imaging system (LI-COR, Biosciences, Lincoln, NE, USA) and quantified using the ImageJ software (https://imagej.net/ij/, accessed on 26–27 June 2017).

### 4.6. Statistical Analysis

Statistical analyses were performed using IBM SPSS statistics 24 (IBM, Amonk, NY, USA), STATISTICA Version 10 (Tibco, Palo Alto, CA, USA), GraphPad Prism (version 10.1.0), and the R-package (version 3.3.3). The differences between the two groups were assessed using the Mann–Whitney U Test. The Kruskal–Wallis Test was used to compare three or more groups. For correlation analyses, the Spearman test was used. *p*-values < 0.05 were considered statistically significant.

## 5. Conclusions

Collectively, the current study indicates that high MUC5AC expression and low MUC5B expression in goblet cells in the large airways are associated with cigarette smoke exposure and with chronic bronchitis and emphysema. High MUC5AC and low MUC5B immunohistochemical expression at baseline were also associated with the progression of chronic airflow limitation over time. Thus, we speculate that MUC5AC and MUC5B might be two distinct biomarkers. MUC5B, but not MUC5AC, was positively correlated with expiratory air flows, diffusing capacity, and the dyspnoea score. These results suggest that the MUC5B expression in goblet cells might be a biomarker of lung disease. In vitro experiments and larger clinical studies are warranted to determine whether MUC5AC and MUC5B play a causal role in the pathophysiology of chronic airflow limitation and if they possibly could represent potential therapeutical targets. Future prospective studies may also focus on exploring these mucins’ biomarker potential.

## Figures and Tables

**Table 1:** Correlations between MUC5B expression in goblet cells and expiratory air flows, diffusing capacity, and dyspnoea score at baseline.

Dependent Variable	Valid N	Spearman R	*p*-Value
FEV_1_ (% predicted)	89	0.249	0.018
FVC (% predicted)	89	0.250	0.018
VC (% predicted)	89	0.250	0.018
FEV_1_/FVC (%)	89	0.110	0.304
FEV_1_/VC (%)	89	0.165	0.123
Diffusing capacity (% predicted)	84	0.336	0.002
Dyspnoea score	87	0.272	0.011

Spearman test in all. Values of % predicted were counted according to ECCS reference equations. The dyspnoea score was assessed by Chronic Respiratory Disease Questionnaire. Abbreviations: FEV_1_: forced expiratory volume in 1 s; FVC: forced vital capacity; N: number of observations; VC: slow vital capacity.

**Table 2:** Individuals included in the study at baseline (*n* = 118). FEV_1_ and FVC percentage of predicted (% predicted) values were calculated using ECCS reference equations. Measurements were done at the baseline visit.

Baseline Study Cohort	Never-Smokers	Smokers	Smokers with COPD	Ex-Smokers with COPD
Subjects (n)	40	40	27	11
Female subjects (n (%))	20 (50%)	20 (50%)	12 (44%)	6 (55%)
Age (years)	59.5 (51.0–63.8)	53.0 (49.0–58.6)	60.4 (56.0–63.0)	63.0 (54.1–65.0)
Smoking history (pack-years)	0 (0–0)	34 (29–40)	42 (36–48)	30 (20–38)
Postbronchodilator FEV_1_ (% predicted)	119 (111–127)	111 (103–120)	76 (73–82)	86 (77–96)
Postbronchodilator FVC (% predicted)	121 (110–128)	113 (107–124)	105 (94–110)	104 (95–116)
Postbronchodilator FEV_1_/FVC ratio (%)	81 (77–85)	78 (75–81)	62 (56–65)	64 (55–67)
Sensitive CRP (g/L)	0.89 (0.58–1.80)	1.20 (0.61–2.55)	1.70 (0.69–5.40)	2.00 (0.89–3.80)

Data are presented as n or median (interquartile range). Abbreviations: COPD: chronic obstructive pulmonary disease; FEV_1_: forced expiratory volume in 1 s; FVC: forced vital capacity; CRP: C-reactive protein.

**Table 3:** Individuals included in the follow-up study (*n* = 74). FEV_1_ and FVC percentage of predicted (% predicted) values were calculated using ECCS reference equations. Measurements were done at the follow-up visit (median follow-up time was 8.4 years).

Follow-Up Study Cohort	Never-Smokers	Smokers	Smokers with COPD	Ex-Smokers with COPD
Subjects (n)	30	23	14	7
Female subjects (n (%))	15 (50%)	15 (65%)	6 (43%)	4 (57%)
Age (years)	69.6 (59.6–72.7)	61.2 (57.7–66.0)	68.8 (65.9–71.6)	62.8 (61.2–72.7)
Smoking history (pack-years)	0 (0–0)	40 (35–47)	47 (40–55)	30 (20–37)
Postbronchodilator FEV_1_ (% predicted)	106 (98–111)	101 (88–111)	62 (56–77)	74 (62–90)
Postbronchodilator FVC (% predicted)	108 (99–118)	117 (103–126)	100 (91–108)	103 (96–112)
Postbronchodilator FEV_1_/FVC ratio (%)	79 (72–81)	72 (66–76)	49 (46–58)	62 (55–67)
Sensitive CRP (g/L)	1.10 (0.55–1.90)	1.40 (0.89–2.20)	2.30 (1.10–3.80)	2.20 (1.20–4.40)

Data are presented as n or median (interquartile range). Abbreviations: COPD: chronic obstructive pulmonary disease; FEV_1_: forced expiratory volume in 1 s; FVC: forced vital capacity; CRP: C-reactive protein.

## Data Availability

The data that support the findings of this study are available upon request from the corresponding author. The data are not publicly available due to privacy and ethical restrictions.

## Supplementary Materials

The following supporting information can be downloaded at: https://www.mdpi.com/article/10.3390/ijms252413653/s1.

## Abbreviations

BW	bronchial wash
COPD	chronic obstructive pulmonary disease
CRP	C-reactive protein
CT	computed tomography
FEV_1_	forced expiratory volume in 1 s
FEV_1_/FVC	forced expiratory volume in 1 s/forced vital capacity
FVC	forced vital capacity
MUC	mucin
