# Supplementary material for: Expression Levels of MUC5AC and MUC5B in Airway Goblet Cells Are Associated with Traits of COPD and Progression of Chronic Airflow Limitation
Source: Int J Mol Sci. 2024 Dec 20;25(24):13653. doi: 10.3390/ijms252413653 (PMC11678853; doi:10.3390/ijms252413653)
Supplement: Supplementary file 1 [file ijms-25-13653-s001.zip › Suppl Table S1.pdf]

Supplementary Table S1.

List of antibodies used in the immunohistochemical stainings.

**Antibody**	**Producer| Clone**	**Kit**	**Antigen retrieval**	**Dilution**
MUC5AC	Novocastra/Clone CLH2	Envision	Citrate pH 6	1/ 200
MUC5B	Santa Cruz/Clone H-300	Envision	Citrate pH 6	1/ 1000
