# Supplementary figures and images for: Expression Levels of MUC5AC and MUC5B in Airway Goblet Cells Are Associated with Traits of COPD and Progression of Chronic Airflow Limitation
Source: Int J Mol Sci. 2024 Dec 20;25(24):13653. doi: 10.3390/ijms252413653 (PMC11678853; doi:10.3390/ijms252413653)

A

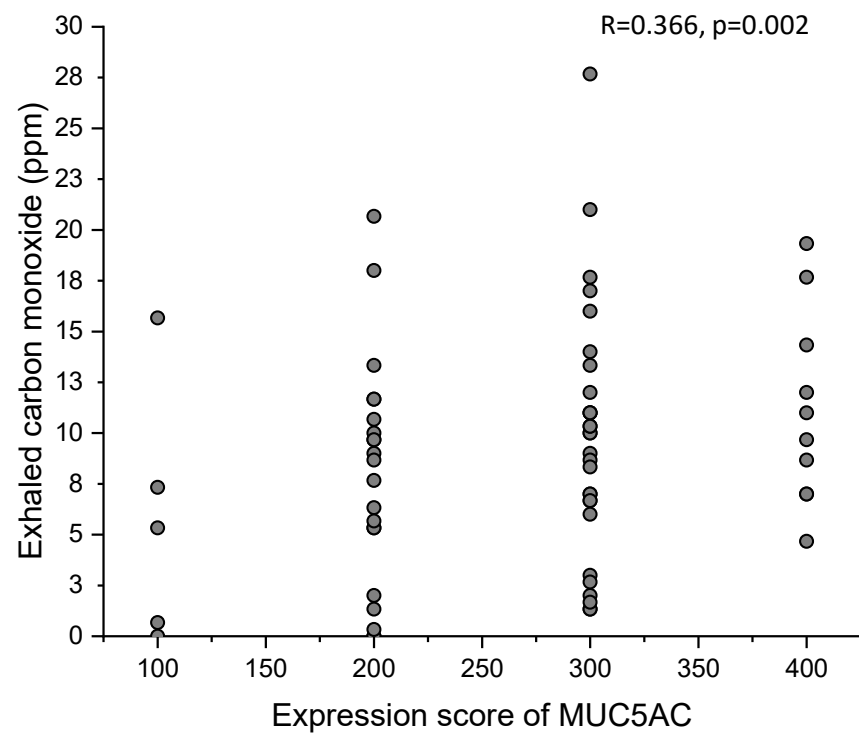

B

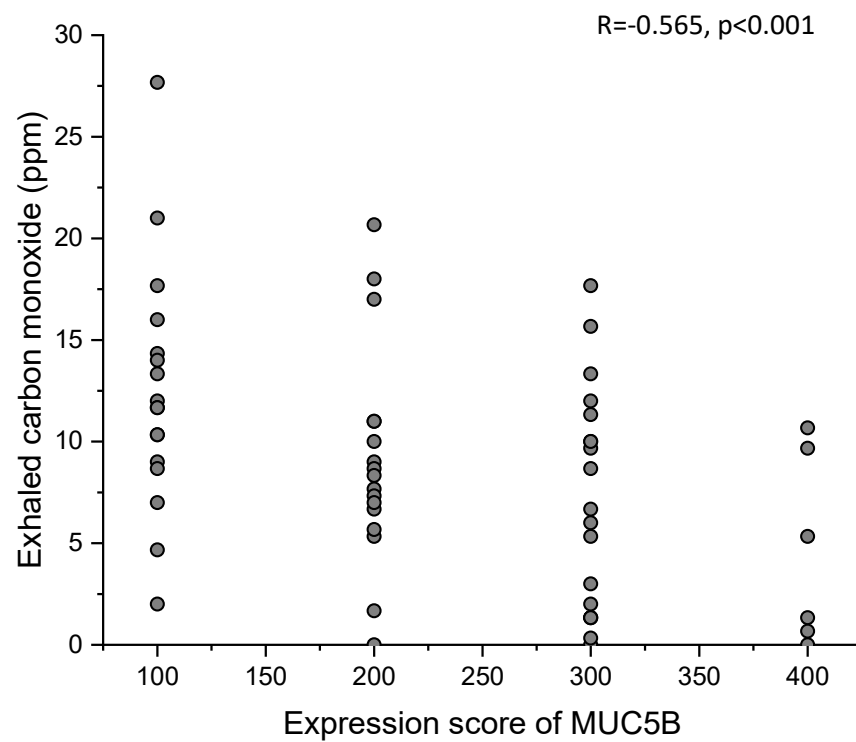

Supplement: Supplementary file 1 [file ijms-25-13653-s001.zip › Suppl Fig S1.pdf]

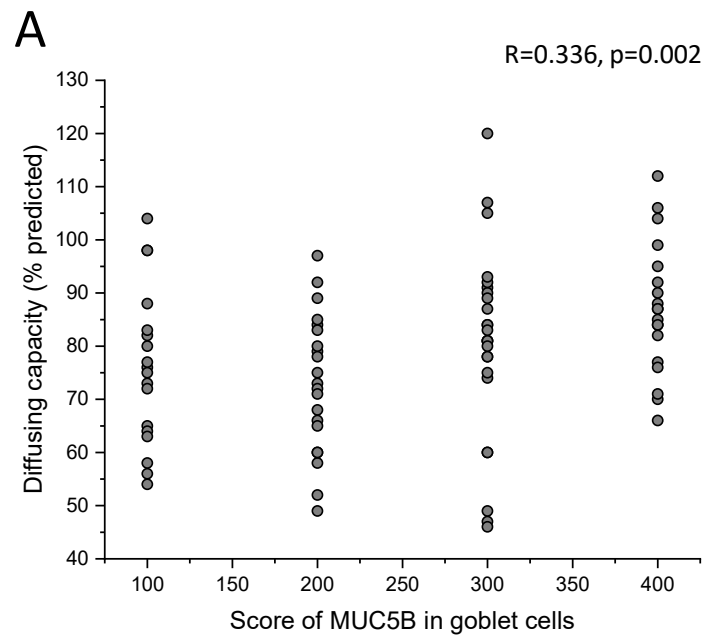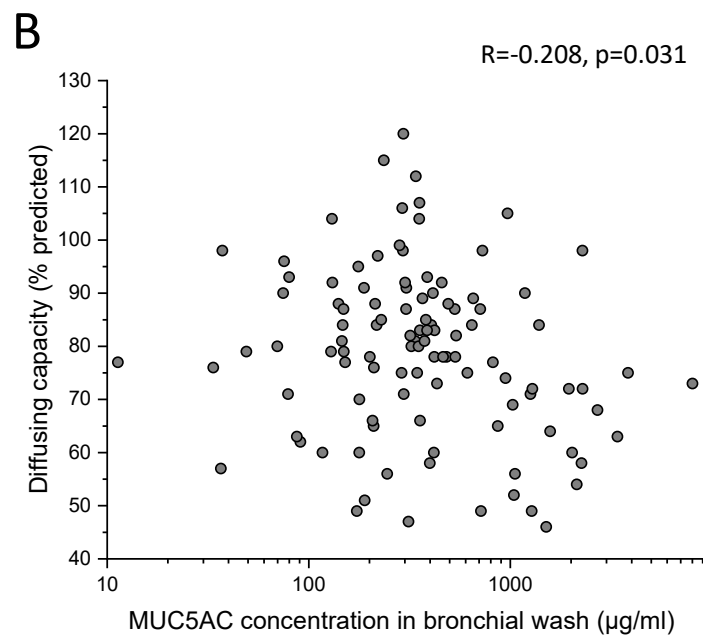

Supplement: Supplementary file 1 [file ijms-25-13653-s001.zip › Suppl Fig S2.pdf]

A

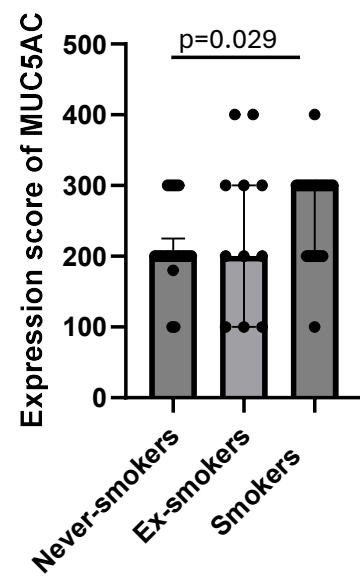

B

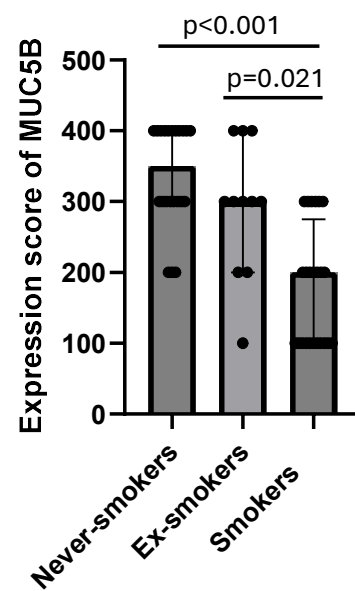

C

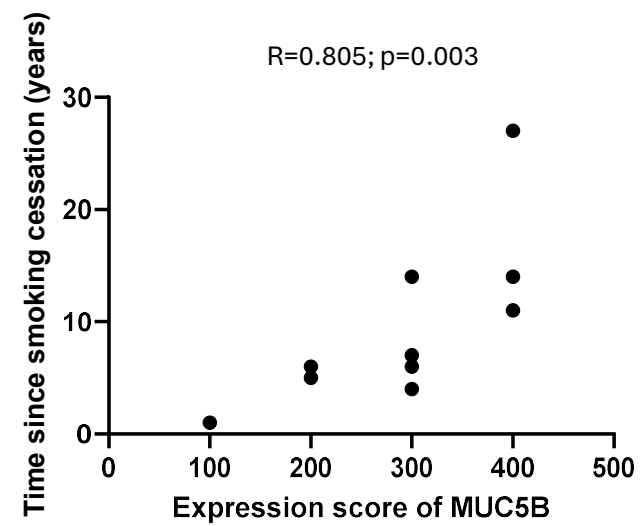

Supplement: Supplementary file 1 [file ijms-25-13653-s001.zip › Suppl Fig S3.pdf]
